# Identification of Antifungal H^+^-ATPase Inhibitors with Effect on Plasma Membrane Potential

**DOI:** 10.1128/AAC.00032-17

**Published:** 2017-06-27

**Authors:** Lasse Kjellerup, Sandra Gordon, Karen O'Hanlon Cohrt, William Dalby Brown, Anja Thoe Fuglsang, Anne-Marie L. Winther

**Affiliations:** a Pcovery, Copenhagen, Denmark; b Department of Plant and Environmental Sciences, University of Copenhagen, Frederiksberg, Denmark

**Keywords:** ATPase, antifungal agents, drug development, membrane proteins

## Abstract

The plasma membrane H^+^-ATPase (Pma1) is an essential fungal protein and a proposed target for new antifungal medications. The compounds in a small-molecule library containing ∼191,000 commercially available compounds were screened for their ability to inhibit Saccharomyces cerevisiae plasma membranes containing Pma1. The overall hit rate was 0.2%, corresponding to 407 compounds. These hit compounds were further evaluated for ATPase selectivity and broad-spectrum antifungal activity. Following this work, one Pma1 inhibitor series based on compound 14 and analogs was selected for further evaluation. This compound series was able to depolarize the membrane and inhibit extracellular acidification in intact fungal cells concomitantly with a significant increase in intracellular ATP levels. Collectively, we suggest that these effects may be a common feature of Pma1 inhibitors. Additionally, the work uncovered a dual mechanism for the previously identified cationic peptide BM2, revealing fungal membrane disruption, in addition to Pma1 inhibition. The methods presented here provide a solid platform for the evaluation of Pma1-specific inhibitors in a drug development setting. The present inhibitors could serve as a starting point for the development of new antifungal agents with a novel mode of action.

## INTRODUCTION

Fungal infections affect 25% of the population, with superficial infections of the skin and nails representing the most common type. In a survey of women (age 16 years and older, *n* = 6,000), up to 49% had been diagnosed with vulvovaginal candidiasis (VVC), depending upon their ethnic origin, and approximately 20% of those women experienced recurrent VVC in a 12-month follow-up period (1), with a pronounced impact on quality of life. Invasive fungal infections are less common but of much greater concern because they are associated with extremely high mortality rates (20 to 90%) (2). The most common invasive fungal infections are caused by the yeasts Candida and Cryptococcus spp., followed by the molds Aspergillus and *Mucor* spp. Key areas of concern in the treatment of invasive fungal infections with the current antifungal medications include delays in diagnosis and the identification of the specific pathogenic species, intrinsic and acquired drug resistance, inconvenient drug administration, safety, and tolerability issues with prolonged use. For these reasons, there is a major unmet need for new antifungal agents (3).

The fungal plasma membrane H^+^-ATPase has long been recognized to be a promising antifungal target (4–6). The proton pump is essential for fungal growth, as shown by knockout studies (7). The *PMA1* gene encodes the H^+^-ATPase, and the pump is referred to as Pma1. The fungal cell is dependent on Pma1 creating an electrochemical gradient across the plasma membrane, which is used by other transporters to energize the uptake of ions and nutrients. Pma1 pumps protons from the cytosol to the exterior of the cell, energized by ATP hydrolysis. In this regard, fungal cells are fundamentally different from human cells, where the plasma membrane potential is created by the Na^+^,K^+^-ATPase (8).

Pma1 belongs to the type III family of P-type ATPases. The related human ATPases, Na^+^,K^+^-ATPase, Ca^2+^-ATPase (sarcoplasmic endoplasmic reticulum Ca^2+^-ATPase [SERCA]), and H^+^,K^+^-ATPase, belong to the type II family. All mammalian ATPases share less than 30% amino acid sequence identity with Pma1. In contrast, the fungal H^+^-ATPase appears to be relatively conserved across the fungal kingdom (the amino acid sequence similarity is generally 70 to 90%). The high level of conservation seen for Pma1 warrants efforts to identify a specific Pma1 inhibitor with broad-spectrum antifungal activity.

Within the last 35 years, a number of nonspecific compounds have been evaluated as Pma1 inhibitors. To date, only a few Pma1 inhibitors, such as ebselen and the peptide BM2, have been demonstrated to inhibit the growth of living fungal cells at concentrations in the low micromolar range (4, 5, 9–12). Omeprazole is an inhibitor of the human H^+^,K^+^-ATPase and has also been evaluated as an inhibitor of Pma1 (4). Studies have shown that single mutations in the proposed binding site in Pma1 greatly alter the growth-inhibitory effects of omeprazole (4). Omeprazole requires an acid activation step to inhibit Pma1, and fungal growth inhibition is pH dependent. For full growth inhibition of Saccharomyces cerevisiae, 430 μM omeprazole is required at pH 3 (4), making it a low-potency antifungal compound.

The classical P-type ATPase inhibitor, vanadate, does not inhibit living cells due to a lack of membrane penetrability. Ebselen is known to inhibit the mammalian H^+^,K^+^-ATPase (13) and the Ca^2+^-ATPase ATP6 of Plasmodium falciparum (PfATP6), in addition to Pma1 (14). Due to the reactivity of ebselen with protein thiols, it is believed to target several enzymes and modify a range of biological activities (15).

Natural products, such as tellimagrandin II (16) and the immunoprotein lactoferrin (17), inhibit Pma1. Tellimagrandin II potently inhibits the growth of S. cerevisiae but not that of Candida albicans. Lactoferrin exhibits antifungal activity against both S. cerevisiae and C. albicans at concentrations in the low micromolar range.

BM2 is a cationic peptide which potently inhibits Pma1 in an ATP hydrolysis assay (50% inhibitory concentration [IC_50_] = 0.5 μM) (10). It displays greater than 90% fungal growth inhibition at 0.63 μM and pH 7.3 and at 5 μM and pH 7.0. At concentrations above the MIC for S. cerevisiae, BM2 also inhibits the Na^+^,K^+^-ATPase and HEp-2 cell growth and causes blood cell lysis. BM2, ebselen, and vanadate have been selected as comparators to the compounds characterized in this work.

In the present study, selected compounds were screened for their ability to inhibit ATP hydrolysis by measuring the formation of free phosphate ions, corresponding to inhibition of Pma1 under the assay conditions used. The identified inhibitors were tested for their effects on the membrane potential, the intracellular ATP concentration, extracellular medium acidification, and fungal growth inhibition.

## RESULTS

### Screening and evaluation of 191,000 compounds for Pma1 inhibition.

**FIG 1:**
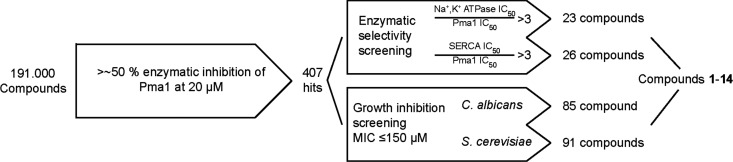
Overview of library screening. Hits were tested for selectivity for the two mammalian ATPases SERCA and Na^+^,K^+^-ATPase as well as antifungal activity against Candida albicans and Saccharomyces cerevisiae. From this, several promising Pma1 inhibitors were identified, with compound 14 being the most promising candidate.

A large library of small-molecule compounds was employed with the aim of finding novel Pma1 inhibitors. In total, 191,000 library compounds were screened using the ATP hydrolysis assay. An overview of the screening cascade is presented in Fig. 1. Compounds (at a final concentration of 20 μM) inhibiting Pma1 enzymatic activity by greater than 50% were regarded as hits in this study. All 407 hits were then counterscreened for their activity against Na^+^,K^+^-ATPase and SERCA in order to distinguish Pma1-specific compounds from general P-type ATPase inhibitors. All hits were screened for potential antifungal activity against S. cerevisiae and C. albicans.

**FIG 2:**
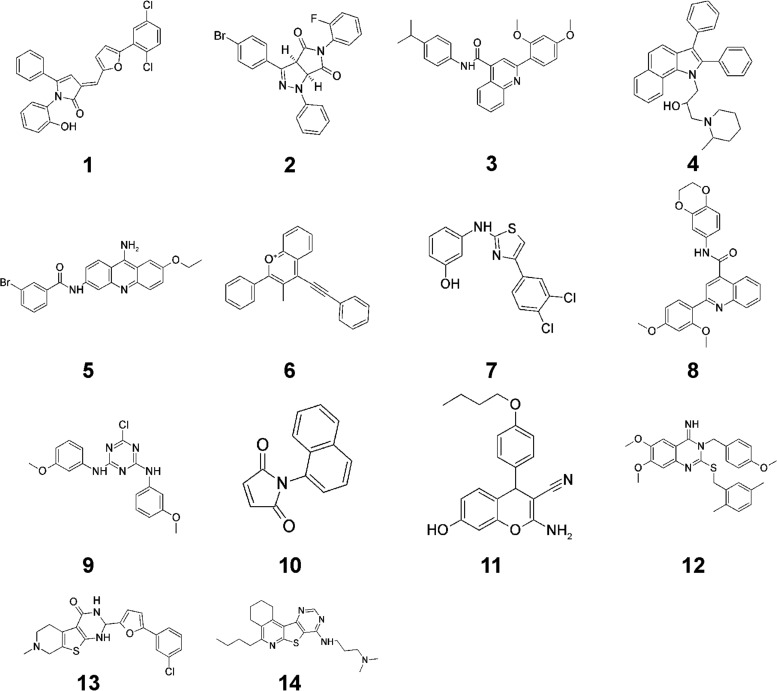
Structures of selected Pma1 inhibitors identified in the library screening.

After evaluating the 407 hits, a selection of compounds was repurchased and reevaluated for Pma1 inhibition and antifungal activity. These steps were undertaken to confirm the structural identity of the library compounds and to ensure that no compound degradation had occurred during the library storage period. The structures of a selected number of these hits are presented in Fig. 2.

### Evaluation of Pma1 inhibitors.

**FIG 3:**
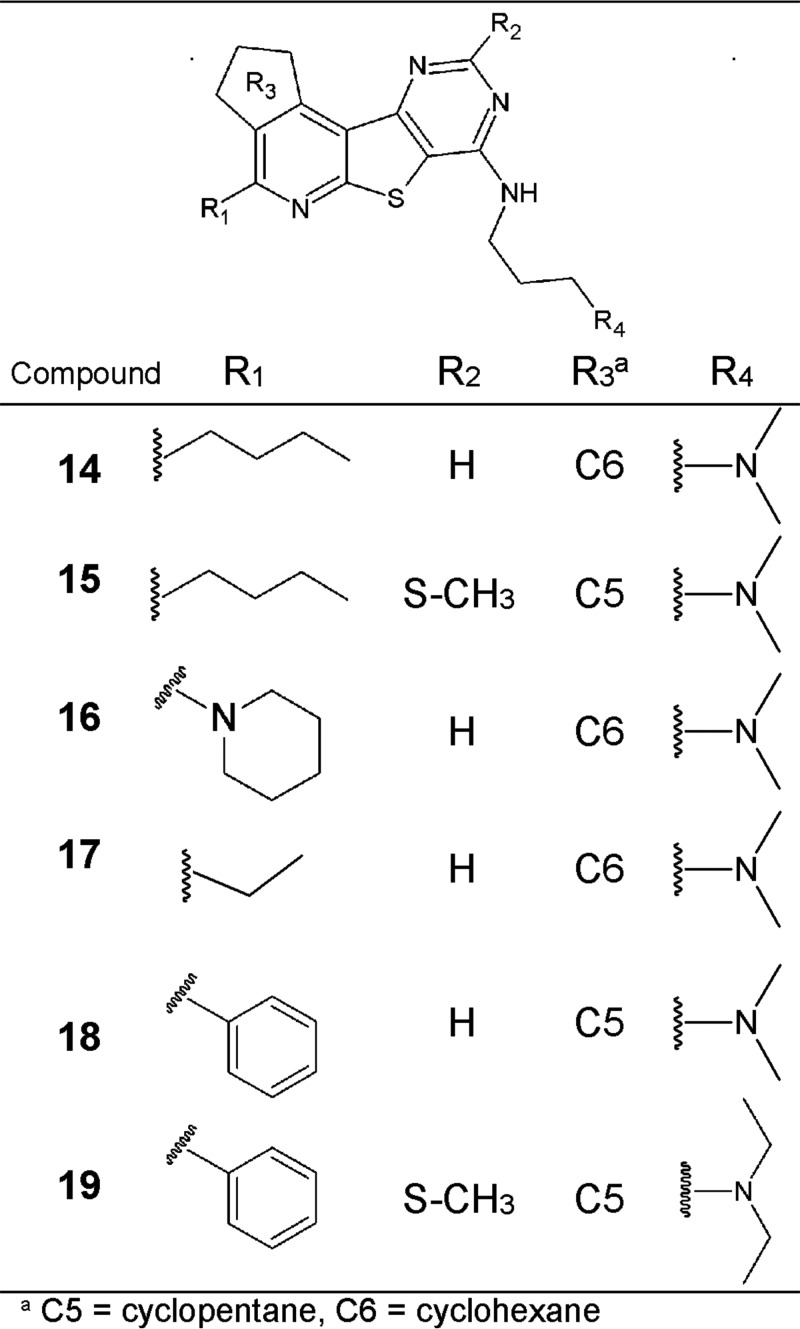
Structure of compound 14 and analogs.

Several interesting compounds with Pma1-inhibitory activity (IC_50_ < 25 μM) were identified through the library screening (Table 1). However, a number of these compounds, compounds 1, 2, 3, 6, 9, 11, and 12, were poor antifungal compounds, with their MICs being above 150 μM for several of the Candida spp. tested (Table 1). Compounds 4 and 8 were also poor antifungal compounds, with their MICs being >38 μM, which was the highest concentration tested due to poor compound solubility in dimethyl sulfoxide (DMSO). The precipitation of several of these compounds observed in the fungal growth medium may partially explain their lack of antifungal activity (Table 1). Unfortunately, several of these compounds that lacked antifungal activity were the compounds that were selective for Pma1 rather than SERCA and Na^+^,K^+^-ATPase. Compounds 5, 7, 10, and 13 were all potent Pma1 inhibitors (IC_50_ < 25 μM) with antifungal activity but were generally more potent against the mammalian ATPases than against Pma1 or equally potent against both the mammalian ATPases and Pma1. The most promising hit from the screen was compound 14, a pyrido-thieno-pyrimidine, whose structure is shown in Fig. 3, together with the structures of selected analogs of compound 14. Compound 14 was the only compound more potent against Pma1 than against the mammalian ATPases and which displayed a broad spectrum of antifungal activity against both yeasts and molds (Table 1).

**TABLE 1:** ATP hydrolysis data and growth inhibition for identified Pma1 inhibitors from the library screening^*a*^

Compound	IC_50_ (μM)			MIC (μM)				
	Pma1	Na^+^,K^+^-ATPase	SERCA	C. albicans	C. parapsilosis	C. tropicalis	C. glabrata	A. fumigatus
1^*b*^	0.040 ± 0.035	13.2 ± 0.91	17.5 ± 4.7	>150^*c*^	>150	>150	>150	>150
2	2.1 ± 1.3	>105	>105	>150^*d*^	NA	NA	NA	NA
3	2.5 ± 2.5	>105	>105	>150^*e*^	>150	>150	>150	>150
4^*b*^	3.7 ± 0.7	15.0 ± 7.2	4.1 ± 0.2	13^*d*^	>38	12	>38	>38
5^*b*^	4.4 ± 0.6	1.3 ± 0.3	0.46 ± 0.04	6.5^*f*^	7.5	7.5	5.8	>75
6^*b*^	5.0 ± 3.0	3.5 ± 3.7	3.4 ± 0.6	>150^*d*^	>150	>150	>150	>150
7^*b*^	5.5 ± 0.3	5.8 ± 0.8	3.8 ± 0.6	7.3^*d*^	8.2	26	4.7	>150
8	6.9 ± 2.0	>105	>105	>38^*d*^	>38	>38	>38	>38
9	9.2 ± 1.0	>105	>105	>150^*d*^	>150	>150	>150	>150
10^*b*^	9.4 ± 1.7	>105	3.4 ± 1.8	88	116	15	12	99
11	12.8 ± 5.0	30.3 ± 2.4	15.7 ± 2.0	>150^*d*^	>150	>150	>150	>150
12	15.5 ± 8.1	58.7 ± 40.2	>105	12^*e*^	>150	>150	>150	>150
13	22.6 ± 12.4	30.6 ± 11.1	22.0 ± 3.0	23	47	>150	31	NA
14	13.7 ± 2.0	18.4 ± 2.1	42.0 ± 8.0	36	24	24	41	75

a Structures are shown in Fig. 2. For IC_50_, data are from 2 to 3 experiments. For MICs, data are from 2 to 5 experiments. NA, not available.

b The ATP hydrolysis assay was performed at pH 6.5 for Pma1 and pH 7.4 for Na^+^,K^+^-ATPase and SERCA. For all other compounds, the ATP hydrolysis assay was performed at pH 7 for all ATPases.

c Compound precipitation was observed in the growth medium at concentrations down to 15 μM.

d Compound precipitation was observed in the growth medium at concentrations down to 48 μM.

e Compound precipitation was observed in the growth medium at concentrations down to 150 μM.

f Compound precipitation was observed in the growth medium at concentrations down to 75 μM.

Additionally, 48 commercially available analogs were purchased for this study, and all of these analogs shared the common motif shown in Fig. 3. Eight of these analogs had an IC_50_ of less than 20 μM for Pma1. Compound 14 and four of the most potent or selective commercial analogs were chosen for further characterization (Fig. 3; Tables 2 to 5) in parallel with BM2, vanadate, and ebselen. Compound 17 was chosen as a negative control because it had a scaffold similar to that of compound 14 but weak Pma1 inhibition, probably due to the lack of an important lipophilic interaction in the R_1_ position (ethyl versus *n*-butyl in compounds 17 and 14, respectively).

**TABLE 2:** Effects of compound 14 and analogs on ATPase activity^*a*^

Compound	IC_50_ (μM) for ATP hydrolysis			
	S. cerevisiae Pma1	C. albicans Pma1	Na^+^,K^+^-ATPase	SERCA
14	13.7 ± 2.0	17.0 ± 0.9	18.4 ± 2.1	42.0 ± 8.0
15	5.9 ± 0.2	6.3 ± 0.5	4.1 ± 0.6	4.5 ± 0.0
16	7.8 ± 0.6	9.5 ± 1.0	9.1 ± 1.0	11.1 ± 0.0
17	106.7 ± 18.2	150.7 ± 6.2	>333	>333
18	18.5 ± 4.3	22.9 ± 2.0	85.7 ± 26.6	69.5 ± 6.9
19	7.3 ± 1.0	8.7 ± 0.5	5.5 ± 1.8	4.4 ± 0.3
BM2	1.2 ± 0.1	0.9 ± 0.2	10.4 ± 2.7	1.1 ± 0.0
Vanadate	0.25 ± 0.01	0.23 ± 0.05	0.006 ± 0.001	10.4 ± 2.4
Ebselen	0.92 ± 0.15	0.51 ± 0.04	0.16 ± 0.01	0.12 ± 0.00

a Experiments were performed at pH 7, and standard deviations (*n* = 2) are given. Rabbit Ca^2+^-ATPase (SERCA1a) and porcine kidney Na^+^,K^+^-ATPase were used.

The four most potent analogs had a low level of Pma1 selectivity (Table 2), with compound 18 being the most selective, exhibiting approximately 3- to 4-fold greater selectivity toward Pma1 than toward SERCA or Na^+^,K^+^-ATPase. Compounds 15, 16, and 19 were more potent than compound 14 but had no selectivity for Pma1. All of the compounds exhibited antifungal activity against both Candida and Aspergillus spp. (Table 3), as well as a reduction in their activity on the viability/proliferation of mammalian Hep-G2 cells (Table 4). In our hands, BM2 was 8-fold more selective for Pma1 over Na^+^,K^+^-ATPase inhibition but had no selectivity toward SERCA. Furthermore, BM2 inhibited the growth of S. cerevisiae and C. albicans but had no effect on mammalian Hep-G2 cells.

**TABLE 3:** Antifungal activities of compound 14 and analogs against yeast and mold species

Compound	MIC (μM) for growth inhibition						
	S. cerevisiae	C. albicans	C. parapsilosis	C. tropicalis	C. glabrata	A. fumigatus	A. flavus
14	7.5	36	24	24	41	75	75
15	1.5	4.8	15	47	15	15	24
16	3.2	15	15	28	11	15	47
17	47	150	150	150	150	>150	>150
18	24	75	24	58	41	75	>75
19	3.7	6	3.7	3.7	3.7	12	12
BM2	4.7	15	26	4.7	150	>150	>150
Vanadate	>750	>750	>750	>750	>750	>750	>750
Ebselen	7.5	7.5	24	11	7.5	24	24

**TABLE 4:** Effects of Pma1 inhibitors on Hep-G2 cells

Compound	EC_50_ (μM) for Hep-G2 cells at 24 h
14	2.3 ± 0.0
15	2.8 ± 0.1
16	2.6 ± 0.9
17	5.8 ± 1.1
18	3.6 ± 1.1
19	24.2 ± 0.5
BM2	>100
Vanadate	>500
Ebselen	10.7 ± 7.0

To determine if the observed antifungal activity was caused by Pma1 inhibition or another mechanism, a range of additional assays was performed. Following glucose activation of Pma1, subsequent proton extrusion leads to acidification of the buffer surrounding the cells. Inhibitors which prevent this extracellular acidification likely act through Pma1 inhibition, although these observations alone cannot discriminate between direct or indirect Pma1 inhibition (e.g., via membrane disruption). The acidification of the external medium is measured within a few minutes after compound addition. Thus, compounds which cause fast plasma membrane disruption indirectly affect proton transport and appear to be inhibitors of the acidification process. However, amphotericin B, a marketed antifungal which forms pores in the fungal membrane, did not inhibit extracellular acidification in the assay time frame of 12 min (Table 5). Compound 14 and its analogs inhibited the acidification process at concentrations similar to the IC_50_ for Pma1 in the ATP hydrolysis assay (Tables 2 and 5), which supports the direct inhibition of Pma1. BM2 was slightly more potent in inhibiting the acidification process than inhibiting the purified Pma1 enzyme in the ATP hydrolysis assay (Tables 2 and 5). The IC_50_ of ebselen for C. albicans was found to be 11 μM, in agreement with the previously reported value of 14 μM (18).

**TABLE 5:** Effects of Pma1 inhibitors on medium acidification in S. cerevisiae and C. albicans^*a*^

Compound	IC_50_ (μM) for acidification	
	S. cerevisiae	C. albicans
14	11.2 ± 2.7	6.6 ± 2.5
15	1.7 ± 0.1	1.6 ± 0.0
16	7.6 ± 3.4	3.4 ± 1.6
17	39.6 ± 10.3	19.1 ± 1.7
18	12.5 ± 1.1	8.8 ± 0.4
19	2.3 ± 0.1	2.3 ± 0.5
BM2	0.2 ± 0.0	0.5 ± 0.1
Vanadate	>40	>40
Ebselen	7.6 ± 0.0	9.4 ± 2.1
AMB	>75	>75

a The IC_50_ was determined to be the concentration which resulted in 50% inhibition of medium acidification normalized to the response from glucose-activated versus non-glucose-activated cells. AMB, amphotericin B. IC_50_ with standard deviations (*n* = 2 to 3) are indicated.

### The membrane potential decreases upon Pma1 inhibition without changes in membrane integrity.

A striking feature of the fungal cell membrane is the very high membrane potential generated by Pma1. It has been measured to be −175 mV within the fungal species Neurospora crassa (19). This value is significantly higher than that observed in mammalian cells, where the membrane potential is typically measured to be −65 to −85 mV. The N. crassa measurements were performed on the large hypha of this mold species with patch clamp electrodes, a setup which is technically difficult to reproduce on smaller cells, such as those of S. cerevisiae. To further demonstrate Pma1 inhibition in living fungal cells, image cytometry was employed to investigate membrane potential changes and membrane integrity upon compound exposure. Membrane depolarization was assessed with the fluorescent probe bis-(1,3-dibutylbarbituric acid) trimethine oxonol [DiBAC_4_(3)], which is only able to enter cells with a decreased membrane potential. Simultaneously, propidium iodide (PI) was used to distinguish between membrane depolarization and general membrane permeabilization, since PI only enters cells with permeable cell membranes.

**FIG 4:**
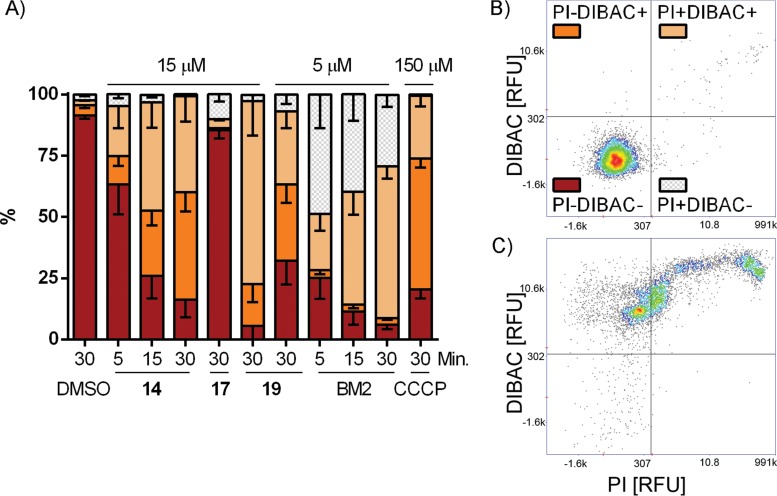
Effects of inhibitors on membrane potential and membrane integrity in S. cerevisiae. (A) Bar chart of the cumulative percentage of cells that were DiBAC_4_(3) positive or negative and PI positive or negative, as defined by the quadrants in panel B. Cells were treated with compound 14, 17, 19, or BM2 or CCCP at 5, 15, or 150 μM for 5, 15, or 30 min (as indicated). Error bars indicating SEMs are shown in only one direction for clarity. (B) Scatter plot of cells treated with 1% DMSO for 30 min (control). (C) Scatter plot of cells treated with 15 μM compound 14 for 30 min. Representative scatter plots for all conditions are shown in Fig. S1 in the supplemental material. RFU, relative fluorescent units.

S. cerevisiae cells exposed to compound 14 yielded an elevated DiBAC_4_(3) signal after 5 min of exposure, while only a minimal increase of the PI signal was observed (Fig. 4A to C and Fig. S1). After 15 or 30 min of exposure, the sizes of both DiBAC_4_(3)-positive cell populations and DiBAC_4_(3)- and PI-positive cell populations had increased. These observations suggest that cells were first depolarized prior to the membrane integrity becoming compromised, thus allowing PI to enter the cells. After 30 min of exposure to compound 14, only 16% of the cells remained both PI and DiBAC_4_(3) negative, 44% were DiBAC_4_(3) positive and PI negative, and 39% were both PI and DiBAC_4_(3) positive. Only 0.7% of the cells were PI positive but DiBAC_4_(3) negative. In the DMSO control sample, 92% of the cells were both PI and DiBAC_4_(3) negative.

The most potent antifungal analog of compound 14 was compound 19, and this compound was also evaluated with imaging cytometry at concentrations of 5 and 15 μM. Similarly to compound 14, exposure of cells to compound 19 also resulted in two large populations of DiBAC_4_(3)-positive and PI-negative cells and DiBAC_4_(3)- and PI-positive cells after 30 min of exposure.

BM2 exposure led to a marked increase in the PI-positive population within 5 min, indicating rapid effects on membrane integrity (Fig. 4A and Fig. S1). However, we also observed an increase in the DiBAC_4_(3) signal after 15 or 30 min in BM2-treated cells. Given the effects on membrane integrity, it is difficult to attribute the increased DiBAC_4_(3) signal to direct Pma1 inhibition. The protonophore carbonyl cyanide *m*-chlorophenylhydrazone (CCCP) had an effect on the PI and DiBAC_4_(3) signals similar to that of compounds 14 and 19. Compound 17, which had an IC_50_ of 107 μM for purified S. cerevisiae Pma1, did not increase the DiBAC_4_(3) signal in S. cerevisiae cells, and the PI signal was not significantly increased.

### iATP levels increase upon Pma1 inhibition.

**FIG 5:**
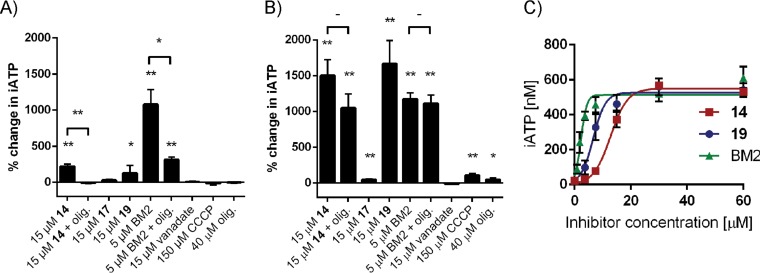
Pma1 inhibitors increase iATP levels in S. cerevisiae (A) and C. albicans (B and C). The total cellular ATP concentrations were determined after 30 min of treatment. The mean data (*n* = 3) with the SD are indicated as the percent change compared to that for the untreated control sample (1% DMSO) (A and B) or as the concentration (in nanomolar) of iATP (C). olig., oligomycin A (at 40 μM in all panels). *, *P* < 0.05 compared to the untreated control by unpaired *t* test; **, *P* < 0.01 compared to the untreated control by unpaired *t* test.

Pma1 requires ATP to energize proton transport out of the fungal cell. A decrease in the intracellular ATP (iATP) levels indirectly affects the activity of Pma1. To rule out the possibility that the Pma1 inhibition observed was indirectly caused by decreased ATP levels, we determined iATP levels after compound exposure (Fig. 5A to C). Treatment of S. cerevisiae and C. albicans cells with compound 14 led to a significant increase in iATP levels (200% and 1,500%, respectively) compared to those for the untreated control. When S. cerevisiae cells were exposed to compound 14 in the presence of oligomycin, an ATP synthase inhibitor, iATP levels remained comparable to those for control cells. Compound 19 behaved in a manner similar to that for compound 14, leading to a significant increase in iATP levels in S. cerevisiae and C. albicans. Treatment with BM2 also led to a large increase in ATP levels (∼1,100%) compared to that for the control cells. When S. cerevisiae cells were treated with BM2 and oligomycin together, the increase in iATP levels was limited to 300%. However, the addition of oligomycin to BM2-treated C. albicans cells did not alter the iATP level compared to that in cells treated with BM2 alone. Treatment with oligomycin alone, CCCP, vanadate, and the low-potency Pma1 inhibitor compound 17 had minimal effects on iATP levels compared to those found after treatment with compounds 14, 19, and BM2. Ebselen could not be tested, as it inhibited the luciferase enzyme used in the iATP assay. The increase in iATP levels in C. albicans for compounds 14, 19, and BM2 was shown to be dose responsive, with all compounds producing a maximum iATP level of about 500 nM (per well) at the highest concentrations of inhibitors tested (Fig. 5C). The half-maximal (50%) effective concentrations (EC_50_) were calculated to 13, 7, and 2 μM for compounds 14, 19, and BM2, respectively. Interestingly, these values were fairly close to the IC_50_ for the Pma1 enzyme from C. albicans of 17, 9, and 0.9 μM, respectively.

## DISCUSSION

In the initial screening process, we identified a number of selective and relatively potent Pma1 inhibitors; however, many lacked antifungal activity. One interesting compound series which exhibited both antifungal activity and an affinity for Pma1 over the mammalian ATPases was identified and further characterized. An initial understanding of the structure-activity relationship for this series was established on the basis of such relationships for commercially available compounds.

The compound 14 series, containing a pyrido-thieno-pyrimidine group, was considered especially interesting since small changes to the scaffold yielded significant changes in the inhibitory properties toward the three ATPases tested. Our data suggest a direct interaction with the ATPases rather than an interaction via compound aggregates, which is a common false-positive mechanism (20, 21). 4-Aminoalkylamino derivatives of pyrido[3′,2′:4,5] thieno[3,2-*d*]pyrimidine have been shown to interact with a number of molecular targets, including phosphodiesterase IV, and to have potential use in the treatment of asthma and chronic obstructive pulmonary disease (22; S. L. M. Pages and M. J. Taltavull, U.S. patent application, June 2006) and in the control of tumor necrosis factor alpha (TNF-α) release (23) and to have beta2 adrenoreceptor agonist activity (24). Additionally, diverse biological activities, such as anticonvulsant (25–27) and neurotropic (28, 29) activities, have been ascribed to compounds similar to those presented here. To our knowledge, this is the first description of pyrido-thieno-pyrimidines as inhibitors of P-type ATPases. The Pma1-inhibitory activity was confirmed both through the inhibition of acidification of the extracellular medium and through the demonstration of membrane depolarization but not an immediate loss of integrity by the DiBAC_4_(3) assay.

DiBAC_4_(3) and PI are frequently used as stains for dead or dying cells. However, DiBAC_4_(3) enters the cells due to a loss of membrane potential, whereas PI enters the cells due to a loss of membrane integrity. By using a short time frame (5 to 30 min) and observing individual cells, DiBAC_4_(3) can be used to estimate changes in membrane potential, while costaining with PI reveals when the membrane loses its integrity. Previously, DiBAC_4_(3) (30) and other fluorescent probes used to measure membrane potential, such as 3,3-dipropylthiacarbocyanide iodide) [diS-C3(3)] (31), have been used to estimate changes in membrane potential. To our knowledge, DMM-11, a competitive inhibitor of Mg^2+^-ATP, is the only other Pma1 inhibitor which has previously been shown to depolarize the fungal plasma membrane (12).

The protonophore CCCP was used as a positive control in the membrane potential study; however, it required high concentrations to overcome the proton excretion induced by Pma1 and depolarize the membrane. CCCP has previously been shown to affect the mitochondrial membrane and, thereby, the supply of ATP (32). In this study, treatment with CCCP resulted in limited changes to the iATP levels in S. cerevisiae and C. albicans (Fig. 5). Treatment with the ATP synthase inhibitor oligomycin did not result in a large significant change in iATP levels, in agreement with earlier observations by Andrés et al. (17). These data suggest that fungal cells can compensate for ATP synthase inhibition for short periods of time under these conditions.

Compounds 14, 19, and BM2 gave rise to a large significant increase in iATP levels, while compound 17 (a low-potency Pma1 inhibitor) gave rise to a substantially lower increase. Increases in iATP levels have previously been reported with exposure to omeprazole (36% increase) in S. cerevisiae (33) and lactoferrin (>600% increase) in C. albicans (17). Both omeprazole and lactoferrin are described to be Pma1 inhibitors, although omeprazole is not fully capable of inhibiting extracellular acidification (33). We suggest that an increased iATP level is attributable to Pma1 inhibition, considering that Pma1 is the major consumer of ATP in the cell and is thought to consume 20 to 50% of the cellular ATP (34). Therefore, unused ATP accumulates in the cell when Pma1 is inhibited. Increased iATP levels have also been observed in Pma1 mutants with partial defects in pumping activity (35), supporting the hypothesis that increased iATP levels are a consequence of Pma1 inhibition.

Ebselen was found to inhibit not only Pma1 but also the mammalian ATPases SERCA and Na^+^,K^+^-ATPase. Given that ebselen has previously been reported to inhibit PfATP6 and the H^+^,K^+^-ATPase (13, 14), this compound is likely an unspecific P-type ATPase inhibitor.

In our hands, BM2 exhibited less selectivity for S. cerevisiae (ATCC 9763) Pma1 over porcine Na^+^,K^+^-ATPase than the previously reported 50-fold selectivity of S. cerevisiae (T48) Pma1 over canine Na^+^,K^+^-ATPase, determined when the IC_50_ of S. cerevisiae (T48) Pma1 and canine Na^+^,K^+^-ATPase were compared (10). Furthermore, we observed no selectivity of BM2 for Pma1 over SERCA. BM2 affected the membrane potential, but given the rapid and severe effects on overall membrane integrity, it is unclear if the depolarization was directly caused by Pma1 inhibition. Increased cell permeation of rhodamine 6G (Rh6G) was also previously observed in an Rh6G efflux assay with >10 μM BM2, which further supports our observations that BM2 compromises membrane integrity (10). Despite the decreased membrane integrity, our results suggest that Pma1 was inhibited, as indicated by the observed large increase in iATP levels.

Oligomycin did not completely prevent the rise in iATP levels of S. cerevisiae cells treated with BM2. Furthermore, iATP levels in C. albicans cells were significantly changed in response to BM2 exposure, regardless of the presence of oligomycin. We therefore speculate that the inhibition of ATP synthase by oligomycin is slower than the accumulation of iATP resulting from the actions of BM2. We speculate that the increase in iATP levels due to Pma1 inhibition, together with BM2's additional membrane-disruptive effects, facilitates the killing of fungal cells.

Together, our data support the suggestion that BM2 operates via a dual mechanism where both the fungal membrane and the fungal proton ATPase are affected, leading to fungal growth inhibition and cell death. Greater selectivity toward SERCA and the Na^+^,K^+^-ATPase is likely required. Special attention should be paid to possible membrane interactions, as BM2 affects fungal membrane integrity and has previously been linked to low-level hemolysis and detectable toxicity in HEp-2 cells (10). On the basis of the membrane potential and integrity results, the compound 14 series has a mode of action different from that of BM2. The antifungal effect of compound 14 appears to be more a direct consequence of Pma1 inhibition as compared to BM2, as the latter affects both Pma1 activity and the membrane integrity. As expected for an inhibitor of the essential and conserved Pma1 protein, the compound 14 series has a broad spectrum of antifungal activity, inhibiting the growth of all Candida and Aspergillus spp. tested. This is in contrast to BM2, which has poor activity against Candida glabrata and the Aspergillus spp.

Some of the compounds identified during the library screen inhibited Pma1 on an enzymatic level; however, this did not always translate into antifungal activity. There are several plausible explanations for this lack of translation. Fungal cells contain a complex cell wall, which may serve as an impermeable barrier to compound entry. The compounds might not be able to cross the cell membrane due to aggregate formation, leading to false-positive hits in the enzymatic assay. False-positive hits may also result from unspecific protein inhibition activity in a homogeneous assay setup (20, 21). Finally, the chemical properties of the compounds must be taken into consideration, as the possibility that some of the compounds tested precipitated when approaching concentrations required for their effect cannot be ruled out.

In this study, novel compounds with Pma1-inhibitory properties were identified and direct inhibition of Pma1 in fungal cells was confirmed by a decrease in the plasma membrane potential, while membrane integrity was preserved. Encouragingly, compounds 14 and 18 displayed selectivity against the mammalian ATPases, with both showing 3-fold selectivity toward SERCA and compound 18 showing 4-fold selectivity toward the Na^+^,K^+^-ATPase. Compound 19 was interesting, as it displayed a 3-fold toxicity window between C. albicans and Hep-G2 cells, despite it being an unselective ATPase inhibitor. However, the fungal growth and Hep-G2 cell assay conditions differed significantly by the presence of 10% fetal bovine serum (FBS) in the Hep-G2 cell assay mixture. In C. albicans growth assays, a 10-fold increase in the MIC value of compound 19 occurred when 10% FBS was added to the assay mixtures (see Table S2 in the supplemental material). This suggests significant protein binding of compound 19. The reduced toxicity window observed when fungal and Hep-G2 cell assays were run under comparable conditions also highlights that selectivity for Pma1 over mammalian ATPases should have high priority in any future Pma1 drug development.

The collection of methods presented herein is especially useful in antifungal drug development targeting Pma1. They can reveal whether inhibition of Pma1 enzymatic activity can be translated into Pma1 inhibition in living cells, while simultaneously ruling out several indirect mechanisms. Future directions for study should include the synthesis of more analogs with the aim of optimizing the potency and specificity of the compounds, thereby improving the toxicity window toward Hep-G2 cells. In conclusion, there is a requirement for improvement of both the physical and chemical properties as well as the selectivity for Pma1 over mammalian ATPases before a new Pma1-targeting drug candidate can be developed.

## MATERIALS AND METHODS

All chemicals were purchased from Sigma-Aldrich (St. Louis, MO) unless stated otherwise. Saccharomyces cerevisiae RS72 was used for purification of the plasma membranes which were used in the library screening. S. cerevisiae (ATCC 9763) was also used for purification of plasma membranes and for all assays except the initial library screening.

### Purification of ATPases.

Plasma membranes containing Pma1 were isolated from S. cerevisiae RS72 cells containing the full-length cDNA of the S. cerevisiae plasma membrane H^+^-ATPase isoform *PMA1* under the control of the *PMA1* promoter, as described in reference 16.

A 100-ml overnight culture in YPD medium [10 g/liter yeast extract (BD, Sparks, MD), 20 g/liter Bacto peptone (BD), 20 g/liter d-(+)-glucose] was transferred to 1 liter YPD medium and grown at room temperature and 150 rpm for 18 h for wild-type S. cerevisiae (ATCC 9763) and 7 h for C. albicans. The cells were harvested and washed in Milli-Q H_2_O by centrifugation at 3,360 × *g*. The cells were glucose activated by the addition of 10% d-(+)-glucose for 10 min while shaking. The cells were then centrifuged at 3,360 × *g* for 3 min and frozen. Cells (60 to 80 g, wet weight) were then disrupted by bead beating (BioSpec, Bartlesville, OK) and processed as described in reference 16 to prepare the plasma membranes. For C. albicans only, microsomes were prepared, as the sucrose gradient step did not provide additional purity. All batches were validated by ATPase hydrolysis activity, pH optimum, and orthovanadate sensitivity.

Rabbit sarcoplasmic reticulum membranes containing sarcoplasmic endoplasmic reticulum Ca^2+^-ATPase (SERCA) were kindly provided by Claus E. Olesen and Jesper V. Møller, Aarhus University, and prepared as described in reference 36. Porcine kidney Na^+^,K^+^-ATPase was kindly provided by Natalya Fedosova, Aarhus University, and prepared as described in reference 37.

### Measurement of ATP hydrolysis.

The protein activity levels were determined by measuring the amount of free phosphate produced from the ATP hydrolysis reaction. The assay was performed as described in reference 16. Each well contained ATPase enzyme, 2 μl of inhibitor (usually in the range of 333 to 0.01 μM) dissolved in dimethyl sulfoxide (DMSO) (Duchefa Biochemie, Haarlem, The Netherlands), and an assay buffer dependent on the enzyme for a final volume of 60 μl. Possible compound precipitation in the assay buffer was monitored using a Nephelostar plate reader measuring turbidity, and the information was taken into account in the IC_50_ determination. Pma1 assay buffer consisted of 20 mM 3-(*N*-morpholino)propanesulfonic acid (MOPS)–NaOH, pH 6.5 or 7.0, 8 mM MgSO_4_, 50 mM KNO_3_ (a vacuolar ATPase inhibitor), 25 mM sodium azide (a mitochondrial ATPase inhibitor), and 250 μM sodium molybdate (an acid phosphatase inhibitor). SERCA buffer consisted of 9 mM MOPS-NaOH, pH 7, 2.7 mM MgCl_2_, 90 μM CaCl_2_, and 72 mM KCl. Na^+^,K^+^-ATPase buffer consisted of 30 mM MOPS-NaOH, pH 7, 40 mM NaCl, 4 mM MgCl_2_, and 20 mM KCl. The concentration of protein was adjusted to obtain a signal of the optical density at 860 nm (OD_860_) of between 0.5 and 1.0 in the untreated samples (for all Pma1 batches, ∼1 to 2 μg/well; for SERCA and Na^+^,K^+^-ATPase, ∼0.1 to 0.2 μg/well). BM2 (RRRFWWFRRR-NH_2_, d-form amino acids) was custom synthesized by Peptide 2.0 (Chantilly, VA).

### Library screening.

Libraries of compounds in a 96-well plate format were screened for Pma1 inhibition with the purified S. cerevisiae RS72 plasma membranes using the ATP hydrolysis assay at pH 6.5. All compounds were diluted in DMSO. The libraries were commercially available from the following suppliers: Key Organics (13,681 compounds; Cornwall, UK), ChemBridge (63,042 compounds; San Diego, CA), ChemDiv Inc. (34,000 compounds; San Diego, CA), InterBioScreen (24,072 compounds; Chernogolovka, Russia), Specs (36,854 compounds; Zoetermeer, The Netherlands), Asinex (9,021 compounds; Rijswijk, The Netherlands), and ComGenex (10,000 compounds; Budapest, Hungary). The CAS numbers of selected compounds are as follows: compound 1, 375352-86-6; compound 2, 845990-59-2; compound 3, 496771-56-3; compound 4, 372174-76-0; compound 5, 488107-35-3; compound 6, 847044-59-1.

### Fungal growth inhibition assay.

The following fungal isolates were used: Saccharomyces cerevisiae ATCC 9763, Candida albicans SC5314, Candida parapsilosis ATCC 22019, Candida tropicalis Ct016, Candida glabrata ATCC 90030, Aspergillus fumigatus ATCC 13073, and Aspergillus flavus ATCC MYA-1005. Frozen stocks of yeast isolates in glycerol (final concentration, 20%) were prepared by growing the cells to log phase in YPD medium at 30°C and 150 rpm. Frozen stocks of mold spores were prepared by harvesting spores from 7-day-old potato glucose agar plates in phosphate-buffered saline, 0.1% Tween 80 and aliquoting these spores in the presence of glycerol (final concentration, 20%). The cells were stored at −80°C. The cells or spores were thawed and diluted to a final concentration of 0.5 × 10^5^ to 2.5 × 10^5^ CFU/ml in sterile Milli-Q H_2_O. In a 96-well plate, the fungal suspension was added to an equal volume of 2× RPMI medium (20.8 g/liter RPMI 1640 medium [catalog number R6504; Sigma-Aldrich], 0.33 M MOPS, 36 g/liter glucose adjusted to pH 7.0 with KOH) to which an inhibitor compound had been added, thus giving a final DMSO concentration of 1.5% in 1× RPMI medium. The plate was incubated for 20 to 24 h at 34°C (72 h for molds), and the OD_490_ was measured on a Victor X5 (PerkinElmer) plate reader. Mold growth assay plates were also visually inspected. The MIC was defined as the lowest concentration inhibiting the visual growth of the microorganism. Standard errors were typically below 5%.

### Hep-G2 cell viability assay.

In a tissue culture-treated 96-well plate (catalog number 655180; Grenier), 10,000 human hepatocyte Hep-G2 cells (catalog number 85011430; Sigma-Aldrich) were plated in 200 μl growth medium (Eagle minimal essential medium; catalog number M2279; Sigma-Aldrich), 2 mM l-glutamine (catalog number 03-020-1B; Biological Industries), 1% nonessential amino acids (catalog number XC-E1154/100; Biosera), 10% fetal bovine serum (catalog number BI-04-007-1A; Biological Industries) and incubated overnight at 37°C in 5% CO_2_. On the next day, fresh growth medium plus 2 μl compound in DMSO was added. The plate was incubated for a further 24 h at 37°C in 5% CO_2_. The medium was replaced with 100 μl freshly prepared 0.5 mg/ml 2,3-bis(2-methoxy-4-nitro-5-sulfophenyl)-5-[(phenylamino)carbonyl]-2*H*-tetrazolium hydroxide, sodium salt, solution (catalog number X4251; Sigma-Aldrich) in RPMI 1640 medium (catalog number R7509; Sigma-Aldrich) with 3.83 μg/ml phenazine methosulfate (catalog number P9625; Sigma-Aldrich) and incubated for 2 to 3 h at 37°C in 5% CO_2_. The color reaction was measured on a Victor X5 plate reader (PerkinElmer) by determination of the OD_450_ and the half-maximal (50%) effective concentration (EC_50_) was calculated.

### Medium acidification assay.

Frozen stocks of C. albicans and S. cerevisiae were transferred to YPD agar plates and incubated overnight at 34°C. On the next day, the cells were suspended and washed twice in 50 mM KCl, pH 6.7, after pelleting of the cells by centrifugation at 3,000 × *g* for 5 min. The cells were then starved overnight at 4°C in the KCl solution. The cell suspension (final OD_600_ of 0.19), an inhibitor in DMSO (1.5%), and 1.3 μg/ml dextran-fluorescein isothiocyanate (molecular weight, 40,000; catalog number FD40S; Sigma-Aldrich) were then mixed before initiation of the assay by the addition of 2% d-(+)-glucose to a final assay volume of 200 μl. The pH drop was then monitored by determination of the fluorescent signal with excitation at 485 nm and emission at 538 nm on a Fluoroskan Ascent plate reader (Thermo Fisher Scientific) for 12 min. The rate of medium acidification was calculated on the basis of the slope of the drop in fluorescence.

### Membrane potential and integrity assay.

Cells were transferred from frozen stocks to 3 ml of YPD medium and grown overnight at 30°C and 150 rpm. The culture was diluted to an OD_600_ of 0.15 and grown for 3 to 4 h at 30°C and 150 rpm to an OD_600_ of 0.5 to 0.7. The cells were pelleted by centrifugation at 2,000 × *g* for 2 min and washed in buffer A (100 mM MOPS and 1 mM KCl adjusted to pH 7.0 with Trizma base). The washing procedure was repeated, and the cells were resuspended in buffer A to an OD_600_ of 0.2. Fifty microliters of buffer A containing 3.3 μg/ml propidium iodide (PI; catalog number P1304MP; Thermo Fisher Scientific) and 1 μg/ml DiBAC_4_(3) (catalog number D8189; Sigma-Aldrich) was mixed with 50 μl the S. cerevisiae cell suspension in buffer A and transferred to a 96-well plate with 1 μl of inhibitor in DMSO, and the components were mixed.

Approximately 30 μl sample was transferred to an A2 glass slide (ChemoMetec, Allerod, Denmark), and the slide was incubated at 30°C before the cells were counted on a NucleoCounter NC-3000 cytometer (ChemoMetec). Five thousand cells were counted per experiment with an exposure time of 1,000 ms. A dark field was used as the masking channel to select the yeast cells. The DiBAC_4_(3) channel (excitation, 530 nm; emission, 675/75 nm) and the PI channel (excitation, 630 nm; emission, 740/60 nm) were used to measure the membrane potential and membrane integrity, respectively. To account for the fluorescent spillover from the PI channel to the DiBAC_4_(3) channel, 15% compensation was applied. In all experiments, 1.9% compensation was used to compensate for the DiBAC_4_(3)-to-PI spillover. The results were analyzed using NucleoView software (ChemoMetec). Only cells with a pixel size of 10 to 40 were included in the analysis, thus avoiding analysis of noncell artifacts. Carbonyl cyanide *m*-chlorophenylhydrazone (CCCP) was used as a positive control for decreased membrane potential, and lysis buffer was used to validate lost membrane integrity.

### Intracellular ATP.

S. cerevisiae and C. albicans cells were grown and washed as described above for the membrane potential and integrity assay and resuspended in SG medium (0.7% yeast nitrogen base without amino acids [BD], 50 mM succinic acid adjusted to pH 7 with Trizma base) to OD_600_ of 0.2 and 0.1, respectively. One hundred microliters of an S. cerevisiae or C. albicans cell suspension and 1 μl compound in DMSO were incubated for 30 min. Twenty-five microliters of the suspension was then transferred to a black 96-well plate containing 25 μl BacTiter-Glo reagent (Promega, Madison, WI) and incubated for 15 min in the dark. The luminescence was read on SpectraMax X5 (Molecular Devices, Sunnyvale, CA) plate reader with a 10 s of shaking and a 0.5 s integration time. Standard curves at 10, 100, and 1,000 nM ATP were performed with every experiment.

## Supplementary Material

Supplemental material
