# Supplementary material for: Identification of Antifungal H+-ATPase Inhibitors with Effect on Plasma Membrane Potential
Source: Antimicrob Agents Chemother. 2017 Jun 27;61(7):e00032-17. doi: 10.1128/AAC.00032-17 (PMC5487681; doi:10.1128/AAC.00032-17)
Supplement: Supplemental material [file supp_61_7_e00032-17__index.html]

Supplemental material 

# Identification of Antifungal H+-ATPase Inhibitors with Effect on Plasma Membrane Potential

## Supplemental material

- Supplemental file 1 -

  Supplemental Figure S1 and Table S2

  PDF, 515K
