# Supplementary material for: Identification of Antifungal H+-ATPase Inhibitors with Effect on Plasma Membrane Potential
Source: Antimicrob Agents Chemother. 2017 Jun 27;61(7):e00032-17. doi: 10.1128/AAC.00032-17 (PMC5487681; doi:10.1128/AAC.00032-17)
Supplement: Supplemental material [file AAC.00032-17_zac007176290s1.pdf]

Supplementary information

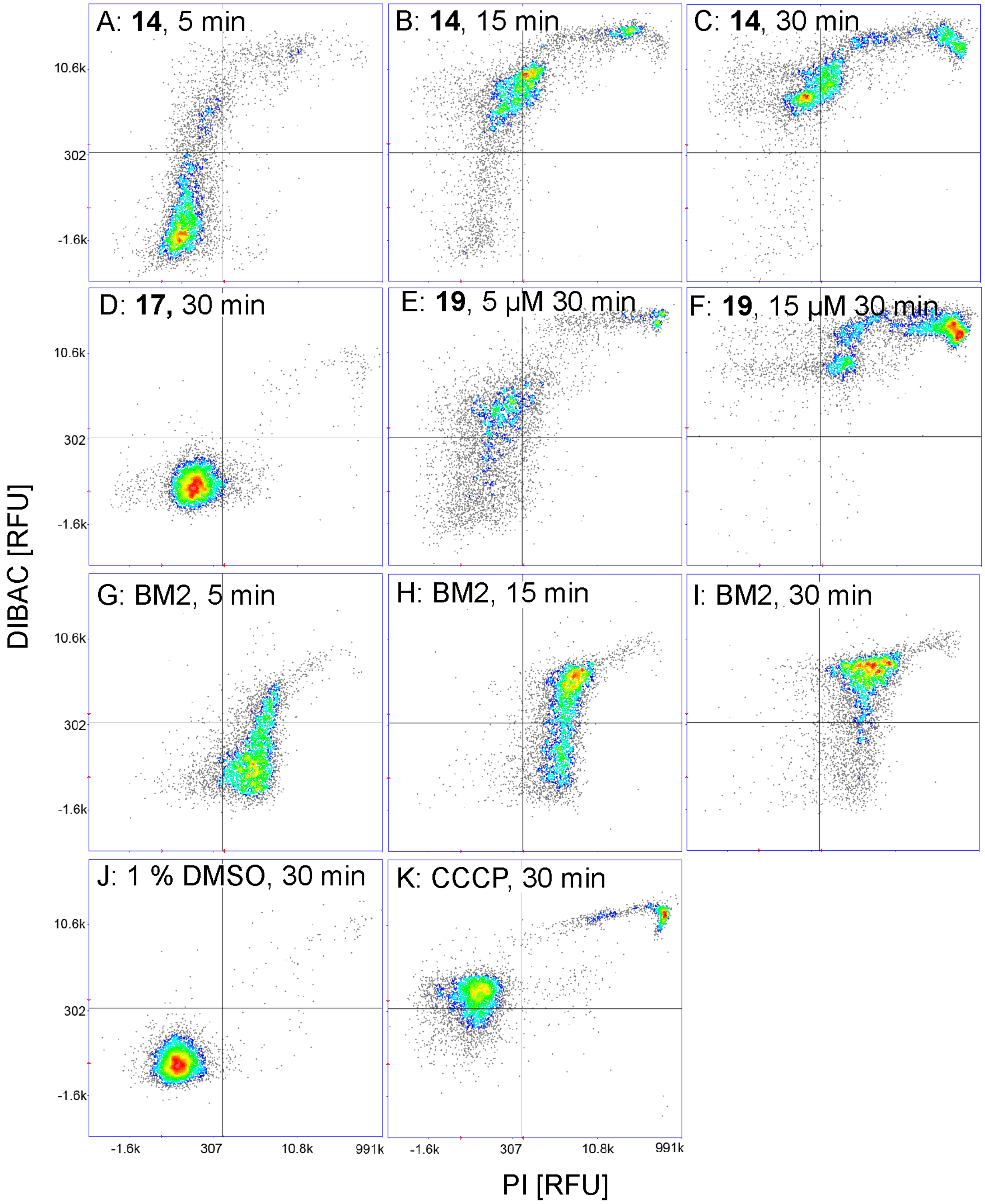

Fig. S1. Effect of inhibitors on membrane potential and membrane integrity in *S. cerevisiae*. Untreated cells (1 % DMSO) remain in the lower left corner. Cells experiencing membrane depolarization exhibit an increased DiBAC signal (y-axis), and cells with decreased membrane integrity exhibited an increased PI signal (x-axis). *Upper row*: Cells treated with compound **1** (15 µM) for 5 (**A**), 15 (**B**) or 30 min (**C**). *Second row:* Cells treated with compound 15 µM **17** (**D**), compound **19** at 5 µM (**E**) or 15 µM (**F**) for 30 min. *Third row*: Cells treated with BM2 (5 µM) for 5 (**G**), 15 (**H**) or 30 min (**I**). *Lower row*: Cells treated with DMSO (1 %, **J**) or CCCP (150 µM, **K**) for 30 min. The 4 quadrants in each graph define cells as DiBAC positive (top quadrants) or negative (lower quadrants) and PI positive (quadrants to the right) or negative (quadrants to the left). The color gradient of gray (lowest) to red (highest) represents cell density. The figures in S1 are representative of the data presented in Fig. 4.

	Growth inhibition (µM)	
	MIC	
	C. albicans **in RPMI**	***C. albicans*** **in RPMI + 10% FBS**
**14**	36	75
**15**	4.8	24
**16**	15	36
**17**	150	150
**18**	75	75
**19**	7	>38
**BM2**	15	36

Table S2. *Candida albicans* growth assay in absence and presence of 10 % FBS. (*n* = 3)
